# Dr. E. K. Kane

**Published:** 1857-05

**Authors:** 


At Havana, on the 16th of February, Elisha Kent Kane, M.D., in the 37th year of his age. Dr. Kane was the eldest son of Judge John K. Kane, of this city. He received his classical education at the University of Pennsylvania, and graduated in its medical department, in the spring of 1843. Prior to his graduation, he served for some months as one of the resident physicians to the Blockley Hospital, at which time he made some researches on the subject of Kiestine, which formed the subject of his inaugural thesis, and being afterwards published, gained him no little reputation. Having received his degree, he entered the navy as assistant surgeon, and in this position he has won a world-wide distinction as a heroic and scientific explorer.

